# Associations of Functional MicroRNA Binding Site Polymorphisms in IL23/Th17 Inflammatory Pathway Genes with Gastric Cancer Risk

**DOI:** 10.1155/2017/6974696

**Published:** 2017-10-08

**Authors:** Kaiyan Dong, Yajuan Xu, Qian Yang, Jiachen Shi, Jicheng Jiang, Yi Chen, Chunhua Song, Kaijuan Wang

**Affiliations:** ^1^Department of Epidemiology and Health Statistics, College of Public Health, Zhengzhou University, Zhengzhou, Henan, China; ^2^Key Laboratory of Tumor Epidemiology of Henan Province, Zhengzhou, Henan, China; ^3^Basic Medical College of Zhengzhou University, Zhengzhou, Henan, China

## Abstract

IL23/Th17 axis acts as an inflammatory pathway in gastric carcinogenesis. MicroRNA- (miRNA-) binding site single-nucleotide polymorphisms (SNPs) of inflammatory genes may alter gastric cancer (GC) susceptibility. In this study, four miRNA binding site SNPs (rs3748067 of *IL17A*, rs887796, rs1468488 of *IL17RA*, and rs10889677 of *IL23R*) were genotyped from 500 patients and 500 controls. Unconditional logistic regression analyses and multifactor dimensionality reduction software were used to evaluate the relationships of SNPs with GC and gene-environment interactions, respectively. Quantitative real-time PCR, Western blot analysis, and luciferase report gene assay were applied for function verification. We found that CT (OR_adj_ = 0.59; 95% CI: 0.44–0.79), CT + TT (OR_adj_ = 0.58; 95% CI: 0.43–0.77) genotypes, and T allele (OR_adj_ = 0.77; 95% CI: 0.47–0.80) of rs3748067 reduced GC risk; the rs10889677 CC genotype (OR_adj_ = 2.22; 95% CI: 1.27–3.87) and C allele (OR_adj_ = 1.24; 95% CI: 1.02–1.52) increased GC risk. A meaningful interaction among ever smoked, family history of GC, and rs3748068 could intensify GC risk by 2.25-fold. Functional tests demonstrated the inhibitory effect of miR-10a-3p on *IL17A* expression in SGC-7901 cells. These results suggested that miRNA binding site SNPs within IL23/Th17 inflammatory pathway genes and their interactions with environmental factors could be associated with GC risk.

## 1. Introduction

Gastric cancer (GC) is a frequently diagnosed cancer with an estimated number of 951,600 new cases and 723,100 deaths worldwide [1]. Despite the declining trends of the incidence and mortality rates, it still ranked as the second leading cause of cancer death among both men and women in China in 2015 [2].

Gastric carcinogenesis is a multifactorial and multistage process. It is accepted that biological factors in which *Helicobacter pylori* (*H. pylori*) infection-induced inflammation account for the main role [3], then a complex network interaction among bacterial factors, host factors, and environmental factors responds to inflammation, and epigenetic changes may also occur and eventually lead to inflammation-related oncogenesis [4]. The T helper 17 (Th17) lineage is a newly discovered subtype of CD4^+^ T cells which is associated with immunity against *H. pylori* infection, and this T cell subset is under the influence of IL23 and produces several proinflammatory cytokines such as IL17A, TNF-*α*, IL17F, IL6, IL21, and IL22 [5, 6]. Also, IL-23 and Th17 correspond to a new axis that drives immune activation and chronic inflammation through the differentiation and activation of Th17 cells which is called the IL23/Th17 axis [7]; the IL23/Th17 axis is a pivotal component of the immune system. Some studies have found that IL17A and IL23R expression were significantly increased in GC tissues and *H. pylori*-related gastritis [8–10]. Accordingly, the IL23/Th17 axis may act as an inflammation pathway in gastric carcinogenesis.

MicroRNAs (miRNAs) could combine to the 3′ untranslated region (3′-UTR) of target messenger RNAs (mRNAs) and interfere with the translation of target proteins in posttranscriptional regulation [11], which means that miRNAs are involved in a number of biological processes. The existence of single-nucleotide polymorphisms (SNPs) in the 3′-UTR region of miRNA target genes might impact on cancer incidence, therapeutics, and prognosis by reinforcing or weakening the expression of miRNA target genes [12, 13]. For example, rs11064 in *TNFAIP8* could function by affecting the affinity of miR-22 binding to the 3′-UTR of *TNFAIP8* and regulating *TNFAIP8* expression, thus contributing to cervical cancer risk [14]; *CXCR2* rs1126579 disrupted a novel binding site for miR-516a-3p, led to a moderate increase in *CXCR2* mRNA and protein expression, and intensified MAPK signaling which reduced the risk of lung cancer [15]. Therefore, we hypothesize that miRNA binding site SNPs in cancer-related genes might associate with the susceptibility and risk of gastric cancer.

At present, great progress about miRNA studies has been made in the field of cancer research, while there are limited mechanism researches about the relationship between miRNA binding site SNPs of inflammatory genes and GC. Our team has recognized series of SNPs within inflammatory genes and their related miRNAs through bioinformatics methods [16], then we have inferred and confirmed the correlations of *IL10RB* and *IL-1* family-related polymorphisms with gastric cancer risk and also preliminarily verified the regulatory effect of miR-197 on *IL1F5* expression [16, 17]. However, the role of miRNA binding site SNPs of IL23/Th17 inflammatory pathway genes on GC risk remains to be evaluated. For this population-based case-control study, four SNPs containing rs3748067 in *IL17A*, rs1468488, rs887796 in *IL17RA*, and rs10889677 in *IL23* were genotyped in 500 GC patients and 500 matched healthy controls. The objective of this study was to examine the associations between the four SNPs with GC susceptibility and test whether miR-10a-3p could regulate *IL17A* by quantitative real-time (qRT) PCR, Western blot analysis, and luciferase report gene assay. Besides, whether interactions between gene-environment factors could contribute to GC was also evaluated.

## 2. Materials and Methods

### 2.1. Study Subjects

We recruited 500 GC patients who were newly diagnosed and histopathologically confirmed for GC, without radiotherapy or chemotherapy previously, and free of other cancers in the First Affiliated Hospital of Zhengzhou University from May 1, 2012, to June 30, 2014. At the same time, 500 cancer-free subjects were selected from a sampling survey of a chronic disease program based on a community in Henan province. The controls were frequency matched with cases by gender and age (±5 years) without evidence of digestive system diseases and were genetically unrelated to the cases. At recruitment, sociodemographic information such as age, gender, smoking status, drinking status, and GC family history had been taken in. Those who had smoked at least one cigarette per day for 1 year and had drunk more than 100 ml of alcohol each time for over 6 months were considered as ever smokers and ever drinkers. All participants signed the informed consents, and the research was approved by the Medical and Health Research Ethics Committee of Zhengzhou University.

### 2.2. Genomic DNA Extraction

Each subject provided 5 ml peripheral blood into a tube containing ethylene diamine tetra acetic acid (EDTA) anticoagulant for DNA extraction. Afterwards, according to the manufacturer's protocol, genomic DNA of the whole blood was extracted by using Tiangen Biotech DNA extraction kit (Beijing Dingguo Changsheng Biotech Co. Ltd., Beijing, China) and then stored at −80°C for genotyping.

### 2.3. SNP Selection and Genotyping

All the potential polymorphisms within miRNA binding sites of IL23/Th17 inflammatory pathway genes were selected with bioinformatics methods: the database SNP (https://www.ncbi.nlm.nih.gov/snp/) was used to search SNPs located in 3′-UTR of interested genes, and the HapMap Project (HapMapRel 28, NCBI B36) was used to screen SNPs according to the criteria of a minor allele frequency (MAF) greater than 0.05 in Chinese Han population. Four SNPs, consisting of rs3748067 in *IL17A*, rs887796, rs1468488 in *IL17RA*, and rs10889677 in *IL23R* were selected for further study. Then, the bioinformatics software packages, including Patrocles database (http://www.patrocles.org/), miRNASNP database 2.0 (http://www.bioguo.org/miRNASNP/), MirSNP (http://cmbi.bjmu.edu.cn/mirsnp), and PolymiRTS database 3.0 (http://compbio.uthsc.edu/miRSNP) were used to find the potential miRNA binding sites of the selected SNPs. Finally, the binding-free energy (kJ/mol) for both common and variant alleles was assessed by RNAhybrid (http://bibiserv.techfak.uni-bielefeld.de/rnahybrid/) and the miRNAs with bigger ΔΔ*G* were chosen (Table 1). The whole experimental processes are presented in Supplementary Figure 1 available online at https://doi.org/10.1155/2017/6974696.

Genotyping of the selected SNPs was performed with polymerase chain reaction-restriction fragment length polymorphism (PCR-RFLP). All primers were designed by the software of Primer 6.0 and synthesized by TaKaRa Biotechnology Co. Ltd. (Dalian, China) as shown in Table 2; the annealing temperature, selected restriction enzymes, and RFLP fragment size were also contained in Table 2. The amplification conditions comprised three stages: firstly, denaturation at 95°C for 5 min; secondly, a total of 35 cycles consisting of denaturation at 94°C for 30 s, annealing at the corresponding temperatures (shown in Table 2) for 45 s, and extension at 72°C for 45 s; finally, an extension step at 72°C for 5 min. The PCR products were checked by electrophoresis on 3.0% agarose gel to ensure successful reproductions. The products after amplification were digested by 5U restriction enzymes (MBI Ferments, St. Leon-Rot, Germany) at the corresponding temperatures for about 10 h. Digested products were visualized to 3.0% agarose gel electrophoresis, and the genotypes were inferred from the number of bands observed in the gel.

In addition, 10% of the DNA samples were selected randomly for direct sequencing (BGI Sequencing, Beijing, China) to confirm PCR-RFLP genotyping results, and the reproducibility was found to be 100% concordant.

### 2.4. Cell Culture and Transfection

The gastric cancer cell lines SGC-7901 and BGC-823 were obtained from the Key Laboratory of Tumor Epidemiology of Henan Province (Zhengzhou, China). These two cell lines were cultured in RPMI-1640 medium supplied with 10% fetal bovine serum in a humidified incubator of 5% CO_2_ at 37°C.

According to the bioinformatics analysis, rs3748067 was located at the binding site of miR-10a-3p in *IL17A*. The miR-10a-3p overexpress vector (GenePharma-miR-10a-3p), negative control vector (NC-vector), miR-10a-3p inhibitor, and its nonspecific control (NC-inhibitor) constructed by GenePharma Co. Ltd. (Shanghai, China) and confirmed by DNA sequencing were transfected into SGC-7901 and BGC-823 cells, respectively, with the Lipofectamine 2000 Reagent (Invitrogen, USA). After 72 h transfection, SGC-7901 and BGC-823 cells were collected for extracting the total RNA and protein.

### 2.5. Quantitative Real-Time PCR Analysis

The relative expression of miR-10a-3p and *IL17A* was examined by qRT-PCR by using the Eco Real-Time PCR System (Illumina, USA). *U6* and *GAPDH* were, respectively, set as reference genes in miR-10a-3p and *IL17A* quantification. Specific stem-loop primers of miR-10a-3p and *U6* were chemically synthetized by TaKaRa Biotechnology Co. Ltd. (Dalian, China). Stem-loop primer sequences are listed as follows: *U6*, GTCGTATCCAGTGCAGGGTCCGAGGTATTCGCACTGGATACGACAAAAATATG; miR-10a-3p, GTCGTATCCAGTGCAGGGTCCGAGGTATTCGCACTGGATACGACTATTCC. The reverse transcription (RT) primer sequences of *U6* and miR-10a-3p are shown as follows: forward primers: CGCAAATTCGTGAAGCGTTC for *U6* and CGGGCCAAATTCGTATCTAGG for miR-10a-3p; consensus reverse primer: CAGTGCAGGGTCCGAGGTAT. The All-in-One qRT-PCR Primer (2 *μ*M) of *GAPDH* and *IL17A* was produced by GeneCopoeia Inc. (Guangdong, China). The qRT-PCR was conducted as the following conditions: an initial denaturation for 20 s at 95°C, then 40 cycles of PCR containing 95°C for 10 s, and 60°C for 30 s. The expression levels of miR-10a-3p and target gene *IL17A*, normalized to *U6* and *GAPDH*, were calculated using the 2^−ΔΔCt^ method and presented as mean ± standard deviation (SD).

### 2.6. Western Blot Analysis

Four groups of proteins were separated on 10% separation sodium dodecyl sulfate (SDS) polyacrylamide gels and 5% concentration SDS polyacrylamide gels, and then transferred to a nitrocellulose membrane which was stained by Ponceau stain and followed by blocking the nonspecific binding site, using 5% skimmed milk. Immunoreactive bands were probed with primary antibody (IL17A, PTEN antibody and internal GAPDH antibody, ABclonal Biotech Co. Ltd., Wuhan, China) and secondary antibody (goat anti-rabbit IgG, Com Win Biotech Co. Ltd., Beijing, China), then visualized via enhanced chemiluminescence. The gray stripes of the Western blot bands were quantitated using ImageJ 1.48v software and corrected by the corresponding internal GAPDH antibody.

### 2.7. Luciferase Report Gene Assay

Six transfection groups including empty vector, *IL17A*-UTR-WT vector, *IL17A*-UTR-WT vector transfected with miR-10a-3p mimic, *IL17A*-UTR-WT vector transfected with negative control mimic (mimic NC), *IL17A*-UTR-WT vector transfected with miR-10a-3p inhibitor, and *IL17A*-UTR-WT vector transfected with nonspecific inhibitor (inhibitor NC) were established to verify the target gene of miR-10a-3p. Luciferase activity was measured with the dual-luciferase reporter assay system (Promega Co. Ltd., Beijing, China).

### 2.8. Statistical Analysis

A statistical package for the Social Sciences version 21.0 (software, Shanghai Co. Ltd., 6761805c6989326cbf14) was used for statistical analyses in this case-control study. The differences between GC and control subjects in the characteristic distributions were compared by Student's *t*-test and chi-square (*χ*^2^) test for continuous and categorical variables, respectively. Hardy-Weinberg equilibrium (HWE) was examined using goodness-of-fit *χ*^2^ test to compare the observed genotype frequencies with the expected ones in controls. Odds ratios (ORs) and 95% confidence intervals (CIs) were calculated by unconditional logistic regression with adjustment for smoking status (never, ever), drinking status (never, ever), and family history of GC (no, yes) to evaluate the associations between SNP genotypes and GC risk. The haplotype analysis was carried out with online SHEsis (http://analysis.bio-x.cn/myAnalysis.php). Multifactor dimensionality reduction (MDR) was applied for determining gene-environment interactions. ORs and *P-*trends were used to estimate the relationships between the number of risk alleles and GC risk for both cases and controls by logistic regression. The results of qRT-PCR, Western blot, and luciferase report gene assay were presented as mean ± SD and analyzed by Student's *t*-test. *P* < 0.05 with two-sided tests was considered statistically significant in the research.

## 3. Results

### 3.1. Characteristics of the Study Subjects

500 GC patients and 500 healthy controls were analyzed in this case-control study. Sociodemographic characteristics of case and control populations are shown in Table 3. No significant difference was found between the two groups about age, gender, and drinking status. However, the proportions of the subjects who were ever smokers (OR = 1.53; 95% CI: 1.20–1.97) and had GC family history (OR = 2.48; 95% CI: 1.75–3.50) in cases were significantly higher than those of controls (*P* < 0.01).

### 3.2. Associations of SNPs with GC Susceptibility

The distributions of genotype and allele frequencies of four interesting miRNA binding site SNPs are listed in Table 4. Goodness-of-fit *χ*^2^ test results showed *P* > 0.05 for all the SNPs, indicating that four SNPs all met the HWE. For rs3748067, the genotype and allele distributions exhibited significant differences between cases and controls; subjects carrying CT (OR_adj_ = 0.59; 95% CI: 0.44–0.79) and CT + TT (OR_adj_ = 0.58; 95% CI: 0.43–0.77) genotypes showed a significant decreased risk for GC than individuals carrying the CC genotype after adjusting for smoking status, drinking status, and GC family history (all *P* < 0.01). In addition, T allele (OR_adj_ = 0.77; 95% CI: 0.47–0.80) reduced GC risk than C allele before and after adjustment (*P* < 0.01). As for rs10889677, individuals carrying the CC genotype (OR_adj_ = 2.22; 95% CI: 1.27–3.87) and C allele (OR_adj_ = 1.24; 95% CI: 1.02–1.52) enhanced the risk of GC than those carrying the AA genotype and A allele, respectively, after adjustment (all *P* < 0.05). However, no statistical meaningful difference was observed between the other two SNPs (rs1468488 and rs887796) and GC risk both before and after adjustment (all *P* > 0.05).

The subjects were further divided into subgroups according to age, gender, smoking status, drinking status, and family history of GC for stratified analysis. As shown in Table 5, rs3748067 had more evident effects on reducing GC risk among men (OR_adj_ = 0.55; 95% CI: 0.40–0.75), individuals aged over 50 years (OR_adj_ = 0.57; 95% CI: 0.41–0.78), ever smokers (OR_adj_ = 0.43; 95% CI: 0.29–0.63), ever drinkers (OR_adj_ = 0.50; 95% CI: 0.31–0.79), and those without family history of GC (OR_adj_ = 0.58; 95% CI: 0.43–0.79) (all *P* < 0.01). Meanwhile, a significant higher risk of GC associated with rs10889677 was found in men (OR_adj_ = 1.29; 95% CI: 1.02–1.65), ever smokers (OR_adj_ = 1.40; 95% CI: 1.05–1.85), and individuals having no GC family history (OR_adj_ = 1.33; 95% CI: 1.06–1.66) (all *P* < 0.05). Additionally, rs887796 only showed meaningful association with increased GC risk in ever drinkers (OR_adj_ = 1.78; 95% CI: 1.12–2.85) (*P* < 0.05).

### 3.3. Analysis of Haplotype

The haplotype analysis was accomplished to find potential haplotype blocks between rs1468488 and rs887796 across *IL17RA*. The haplotype distributions of two groups predicted by SHEsis are shown in Table 6. The results demonstrated that three major haplotypes (haplotypes 1–3) were observed and accounted for almost 99% of all possible blocks in both case and control samples. However, the results showed that no haplotype was associated with GC risk with *P* < 0.05.

### 3.4. Associations of Mutational Locus Numbers with GC Risk for Four SNPs

The number of mutational loci was calculated for each subject and then divided into three groups (Table 7). The mutational locus numbers were recorded as 0 for the wild homozygous genotype, 1 for the heterozygous genotype, and 2 for the homozygous mutant genotype in four SNPs. Final results indicated that the increased number of mutational loci had nothing to do with GC risk.

### 3.5. Gene-Environment Interactions

MDR software was used for analyzing the gene-environment interactions of four SNPs (rs3748067, rs10889677, rs1468488, and rs887796), smoking status, drinking status, and family history of GC; the results are detailed in Table 8. The best model showed that there was an interaction among smoking status, family history of GC and rs3748067 with testing balanced accuracy (TBA): 0.59, and cross-validation consistency (CVC): 9/10. It meant that individuals with smoking history, GC family history, and rs3748067 might have a higher GC risk by 2.25-fold (OR = 2.25; 95% CI: 1.75–2.90) (*P* < 0.01).

### 3.6. GenePharma-miR-10a-3p Enhanced the Expression of miR-10a-3p in SGC-7901 and BGC-823 Cells

**Figure 1:**
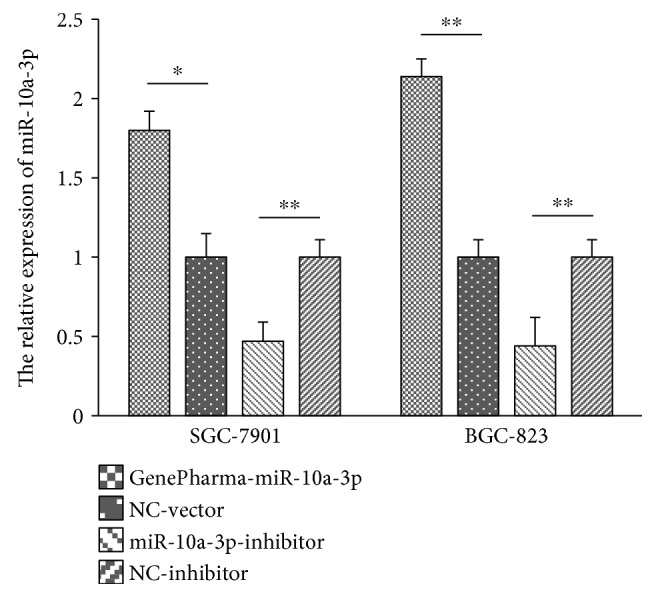
The relative expression of miR-10a-3p after transfection in SGC-7901 and BGC-823 cells. ^∗^*P* < 0.05, ^∗∗^*P* < 0.01. NC-vector: negative control vector; NC-inhibitor: nonspecific inhibitor. miR-10a-3p expression was assessed by qRT-PCR in SGC-7901 and BGC-823 cell lines. Data was evaluated statistically using *t*-test and represent the mean ± standard deviation from the experiments in triplicate.

Total RAN was exacted for reverse transcription after SGC-7901 and BGC-823 cells were transfected with GenePharma-miR-10a-3p, NC-vector, miR-10a-3p inhibitor, and NC-inhibitor; then, the relative expression of miR-10a-3p and internal gene-*U6* was detected by qRT-PCR. The miR-10a-3p relative expression in the GenePharma-miR-10a-3p group (1.80 ± 0.12 for SGC-7901 cells, 2.14 ± 0.11 for BGC-823 cells) was significantly higher than that in the NC-vector group (1.00 ± 0.15 for SGC-7901 cells, 1.00 ± 0.11 for BGC-823 cells) (all *P* < 0.05) (Figure 1), and relative expression of miR-10a-3p was lower in the miR-10a-3p inhibitor group (0.47 ± 0.12 for SGC-7901 cells, 0.44 ± 0.18 for BGC-823 cells) compared with the NC-inhibitor group (1.00 ± 0.11 for both SGC-7901 and BGC-823 cells) (Figure 1) (all *P* < 0.05), which showed that GenePharma-miR-10a-3p and miR-10a-3p inhibitor could, respectively, enhance and decrease the relative expression of miR-10a-3p in both two cell lines. Besides, the results also suggested the successful transfection of GenePharma-miR-10a-3p and miR-10a-3p inhibitor in two cell lines.

### 3.7. GenePharma-miR-10a-3p Downregulated the Expression of IL17A in SGC-7901 Cells

**Figure 2:**
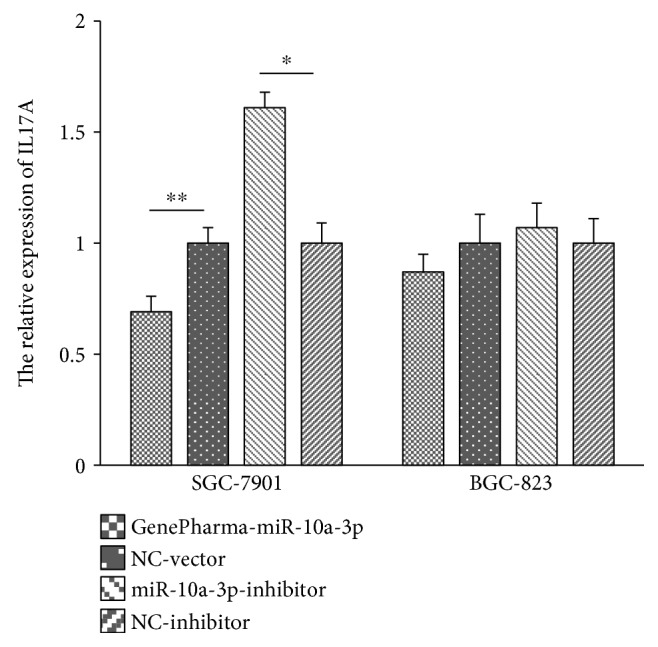
The relative expression of *IL17A* after transfection in SGC-7901 and BGC-823 cells. ^∗^*P* < 0.05, ^∗∗^*P* < 0.01. NC-vector: negative control vector; NC-inhibitor: nonspecific inhibitor. *IL17A* mRNA expression was assessed by qRT-PCR in SGC-7901 and BGC-823 cell lines. Data was evaluated statistically using *t*-test and represent the mean ± standard deviation from the experiments in triplicate.

The relative expression of *IL17A* and internal gene-*GAPDH* was detected by qRT-PCR after GenePharma-miR-10a-3p, NC-vector, miR-10a-3p inhibitor, and NC-inhibitor successfully transfected to SGC-7901 and BGC-823 cells. In SGC-7901 cells, the relative expression of *IL17A* was lower in the GenePharma-miR-10a-3p group (0.69 ± 0.07) comparing with the NC-vector group (1.00 ± 0.07) (*P* < 0.05) (Figure 2), which indicated that GenePharma-miR-10a-3p could downregulate *IL17A* expression in SGC-7901 cells; while compared with the NC-inhibitor group (1.00 ± 0.09), the relative expression of *IL17A* was higher in the miR-10a-3p inhibitor group (1.61 ± 0.07) (*P* < 0.05) (Figure 2), which indicated that *IL17A* expression was upregulated by miR-10a-3p inhibitor in SGC-7901 cells. However, *IL17A* relative expression in the GenePharma-miR-10a-3p group and the miR-10a-3p inhibitor group was not significantly different from their control groups in BGC-823 cells (all *P* > 0.05).

### 3.8. GenePharma-miR-10a-3p Decreased IL17A Protein Expression in SGC-7901 Cells

**Figure 3:**
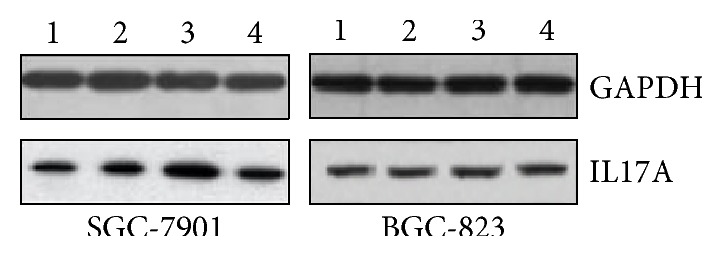
The Western blot stripes of IL17A and GAPDH after transfection in SGC-7901 and BGC-823 cells. The expression of IL-17A protein in SGC-7901 and BGC-823 cell lines was analyzed by Western blot. The protein profiles were normalized with GAPDH antibody. The experiments were independently repeated three times. Line 1: GenePharma-miR-10a-3p. Line 2: NC-vector. Line 3: miR-10a-3p inhibitor. Line 4: NC-inhibitor.

**Figure 4:**
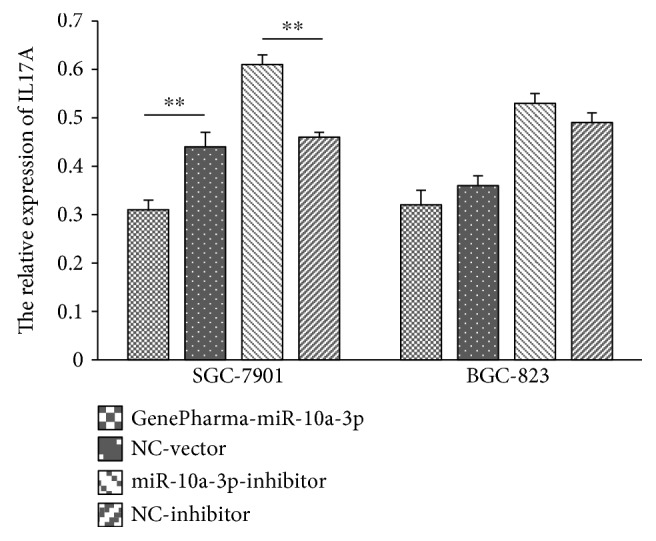
The relative expression of IL17A after transfection in SGC-7901 and BGC-823 cells. ^∗∗^*P* < 0.01. NC-vector: negative control vector; NC-inhibitor: nonspecific inhibitor. IL17A protein expression was assessed by Western blot in SGC-7901 and BGC-823 cell lines. Data was evaluated statistically using *t*-test and represent the mean ± standard deviation from the experiments in triplicate.

Total protein was extracted from four different transfection groups in two cell lines, and the expression of IL17A protein and internal protein-GAPDH was detected by Western blot analysis. The Western blot stripes are presented in Figure 3. In SGC-7901 cells, IL17A protein expression was significantly lower in the GenePharma-miR-10a-3p group (0.31 ± 0.02) compared with the NC-vector group (0.44 ± 0.03) (*P* < 0.05) (Figure 4), while the expression was significantly higher in the miR-10a-3p inhibitor group (0.61 ± 0.02) compared to the NC-inhibitor group (0.46 ± 0.01) (*P* < 0.05) (Figure 4). Besides, consistent with the above results, comparing with the control groups, the GenePharma-miR-10a-3p group and the miR-10a-3p inhibitor group both had no meaningful effect on the expression of IL17A protein in the BGC-823 cell line (all *P* > 0.05). These results obviously revealed that miR-10a-3p could decrease IL17A expression at the protein level only in the SGC-7901 cell line.

### 3.9. miR-10a-3p Regulated the Expression of IL17A-UTR in Luciferase Report Gene Assay

**Figure 5:**
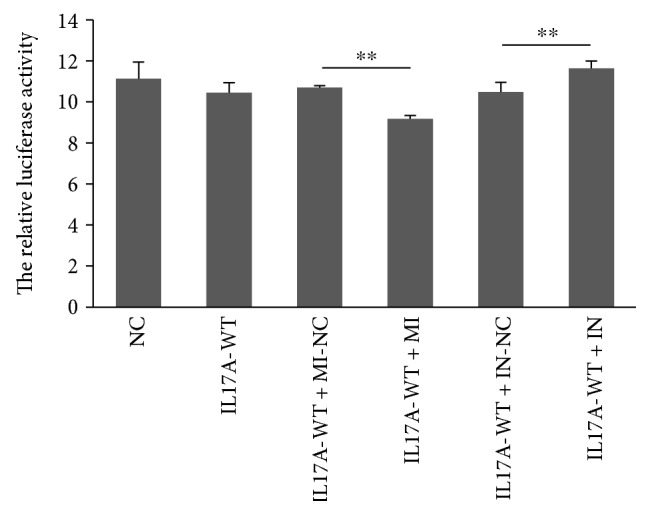
The relative luciferase activity in six groups. ^∗∗^*P* < 0.01. NC: empty vector; IL17A-WT: *IL17A*-UTR-WT vector; IL17A-WT + MI-NC: *IL17A*-UTR-WT vector transfected with negative control mimic; IL17A-WT + MI: *IL17A*-UTR-WT vector transfected with miR-10a-3p mimic; IL17A-WT + IN-NC: *IL17A*-UTR-WT vector transfected with nonspecific inhibitor; IL17A-WT + IN: *IL17A*-UTR-WT vector transfected with miR-10a-3p inhibitor. The relative luciferase was assessed by luciferase report gene assay. Data was evaluated statistically using *t*-test and represent the mean ± standard deviation from the experiments in triplicate.

There were six groups in the luciferase report gene assay: one group for empty vector, one group for single transfection, and four groups for cotransfection (Figure 5). The relative luciferase activity of the empty vector (11.13 ± 0.81) was higher than that of the *IL17A*-UTR-WT vector group (10.45 ± 0.49), while the difference was not statistically significant (*P* > 0.05). The relative luciferase activity in the group that the *IL17A*-UTR-WT vector transfected with miR-10a-3p mimic (9.17 ± 0.17) was lower than that in the *IL17A*-UTR-WT vector group (*P* < 0.05) and also lower when compared with the group that the *IL17A*-UTR-WT vector transfected with mimic NC (10.69 ± 0.09) (*P* < 0.05), which showed that overexpression of miR-10a-3p repressed *IL17A*-UTR. Besides, the relative luciferase activity in the group of the *IL17A*-UTR-WT vector transfected with miR-10a-3p inhibitor (11.63 ± 0.35) was higher than that in the group of the *IL17A*-UTR-WT vector transfected with miR-10a-3p inhibitor NC (10.47 ± 0.48) (*P* < 0.05) and much higher than the group of *IL17A*-UTR-WT vector transfected with mimic (9.17 ± 0.17) (*P* < 0.05), which revealed that the inhibition of miR-10a-3p promoted *IL17A*-UTR expression. All of the results confirmed the negative regulatory role of miR-10a-3p to *IL17A*-UTR.

## 4. Discussion

In this study, we found that miRNA binding site SNPs within IL23/Th17 inflammatory pathway genes were associated with GC risk in Chinese population. The CT genotype (OR_adj_ = 0.59; 95% CI: 0.44–0.79) and T allele (OR_adj_ = 0.77; 95% CI: 0.47–0.80) of *IL17A* rs3748067 were associated with decreased GC risk; the effect was more evident in men (ORadj = 0.55; 95% CI: 0.40–0.75), age > 50 years (ORadj = 0.57; 95% CI: 0.41–0.78), ever smoked (ORadj = 0.43; 95% CI: 0.29–0.63), ever drank (ORadj = 0.50; 95% CI: 0.31–0.79), and without family history of GC (ORadj = 0.58; 95% CI: 0.43–0.79), while individuals carrying the *IL23R* rs10889677 CC genotype (OR_adj_ = 2.22; 95% CI: 1.27–3.87) and C allele (OR_adj_ = 1.24; 95% CI: 1.02–1.52) had higher GC risk. In addition, the gene-environment interaction analysis showed that smoking status, family history of GC, and rs3748067 could intensify GC risk by 2.25-fold (OR = 2.25; 95% CI: 1.75–2.90). Furthermore, the results of qRT-PCR and Western blot revealed that GenePharma-miR-10a-3p downregulated the expression of *IL17A* and IL17A protein in SGC-7901 cells, and luciferase report gene assay affirmed the regulatory relationship.

The *IL17A* gene locates on chromosome 6 and encodes IL17A protein, which is abundantly produced by the Th17 subset of CD4^+^ T cells and acts as a signature cytokine of Th17 cells [18]. Luzza et al. [19] reported that biologically active IL17 increases firstly after *H. pylori* infection, then IL17A stimulates the synthesis of IL-1, IL-6, and TNF-*α* and induces fibroblasts to make matrix metalloproteinases (MMPs) which contribute to mucosal damage. In the past few years, researches focused on the relationship of *IL17A* rs3748067 and GC risk have been performed, while the findings were conflicting. Previously, Arisawa et al. [20] suggested that T allele of rs3748067 was associated with a decreased risk for intestinal GC, which was consistent with our results. But Zhang et al. and Zhu et al. [21, 22], respectively, revealed that the rs3748067 TT genotype increased GC risk. In contrast, Gao et al. [23] showed that there was no association between rs3748067 and GC susceptibility in Chinese population. Unequal sample size and different study subjects may contribute to the inconsistent results. The underlying mechanism involved in the associations between them is not yet clear. We hypothesize that rs3748067 may influence *IL17A* expression by modifying the binding affinity between *IL17* and its corresponding miRNAs.

miRNA could inhibit mRNA translation or induce degradation of mRNA by connecting with its target gene [11]; conversely, variants in 3′-UTR of the target gene might affect the corresponding miRNA. Many researchers have demonstrated that miRNA binding site SNPs would influence the development of cancer: Song et al. [24] found that a miR-29c binding site genetic variant in the 3′-UTR of *LAMTOR3* gene was associated with GC risk; Kang et al. [25] indicated that SNPs in 3′-UTR of docking protein 3 gene (*DOK3*) may affect the expression of *DOK3*, then impact on colorectal cancer by altering the miR-370 binding efficiency. Functional research was carried out in *IL17A* because of the significant association between *IL17A* rs3748067 and GC in our study. According to bioinformatics prediction, miR-10a-3p targets *IL17A*. miR-10a-3p is reported to be downregulated in the kidneys of deep hypothermic circulatory arrest rats and its target genes involve in chemokine pathways [26]. Similarly, we found that miR-10a-3p depressed the expression of *IL17A* at mRNA and protein levels in SGC-7901 cells with qRT-PCR and Western blot analysis, respectively. Luciferase reporter gene assay results also reflected its regulatory effect on *IL17A*-UTR. Besides, the results surprisingly showed that GenePharma-miR-10a-3p and miR-10a-3p inhibitor did not influence the expression of *IL17A* in BGC-823 cells. Since SGC-7901 and BGC-823 cells belong to moderate- and low-degree differential gastric cancer cell lines, respectively, the regulatory role of miR-10a-3p to *IL17A* appeared to be affected by the differentiation degree of gastric cancer cells; the following studies using diverse cancer cell lines are needed to verify the supposition.

The *IL-23R* gene is located on chromosome 1p31 and encodes a subunit of the IL-23 receptor, which acts as important as IL23 since the activity of IL23 is mediated by its binding to IL23R. Zwiers et al. [27] have reported that the *IL23R* rs10889677 variant allele contributed to enhancing *IL-23R* mRNA and protein expression by disturbing the binding capacity of let-7e and let-7f in inflammatory bowel diseases; this mechanism might also make sense in gastric cancer. The CC genotype of rs10889677 displayed an increased risk for gastric cancer in this research. Zhang et al. [28] and Tang et al. [29] also reported that the C allele of rs10889677 had a significantly increased risk for the development of ovarian and bladder cancer in Chinese population. On the contrary, Chen et al. [30] found that CC carriers of rs10889677 had decreased GC risk. The causation of inconsistent results is obscure; more studies are needed to ascertain this relationship.

IL17RA which is encoded by the *IL17RA* gene is a common receptor chain for the IL17 family of ligands. It can transduce IL17 signaling, such as the activation of extracellular-regulated protein kinases (ERK), p38, and nuclear transcription factor *κ*B (NF-*κ*B) [31] to impact on cancer, since mitogen-activated protein kinase (MAPK) and NF-*κ*B signals have important roles at the molecular mechanisms of anticancer [32]. Researches always combined IL17RA with IL17A together to define the relationship of IL17RA to cancer, while Jiang et al. [33] studied IL17RA alone and reported that IL17RA was over expressed in human gastric cancer. Moreover, several studies revealed the associations of *IL17RA* gene polymorphisms with a variety of diseases: a cohort study conducted by Coto et al. [34] discovered the relation of rs4819554 in the promoter region of *IL17RA* to impaired renal function; a statistically significant risk was observed for cervical cancer with the variant allele in *IL17RA* rs879576 in the study of Hardikar et al. [35]. There is no study on the relation of *IL17RA* rs1468488 and rs887796 with GC susceptibility yet. While, with respect to this study, only ever drinkers with rs887796 had higher GC risk; apart from that, no meaningful associations were found between these two polymorphisms and GC. Studies with larger sample size in different population are needed to validate the result.

Gene-environment interactions have been identified in gastric cancer, which means that genetic predisposition factors may interact with environmental risk factors in carcinogenesis [36]; for instance, Gao et al. [37] and Boccia et al. [38] found that the interactions between gene polymorphisms and smoking had potential to affect GC risk in Chinese and Italian populations, respectively. Since the traditional procedures with the fitting logistic regression model could lead to an increase in type II error and a decrease in the power of test; in this study, the gene-environmental interactions were detected by the MDR method which could overcome some limitations of logistic regression [39]. Smoking status, family history of GC, and rs3748067 made up the best model with TBA: 0.59 and CVC: 9/10, indicating that the combination of rs3748067, ever smoked, and GC family history intensified GC risk by 2.25-fold (OR = 2.25; 95% CI: 1.75–2.90). The results demonstrated that miRNA binding site SNPs and its interactions with environmental factors could contribute to GC risk together.

There are several advantages which should be mentioned. To start with, the individual matched case-control study design could control selection bias and information bias to a certain extent. Then, 10% of all the samples were randomly selected to assess the repeatability when genotyping, and the PCR products of three genotypes were also selected randomly for sequencing validation. Finally, qRT-PCR and Western blot experiments were independently repeated 3 times to reduce experimental system error for credible results, and luciferase report vector attested the regulating action of miR-10a-3p to *IL17A* directly, which heightened the credibility of functional verification. Meanwhile, the selected genes were not comprehensive to represent all the IL23/Th17 inflammatory pathway genes; the subjects examined in this study were not stratified with the status of *H. pylori* infection due to conditional restriction, and larger sample studies are greatly needed to confirm the results due to the limitation of research cycle and hospital medical records.

## 5. Conclusions

The current case-control study suggested that *IL17A* rs3748067 CT and CT + TT genotypes, as well as T allele, could decrease GC risk; the rs10889677 CC genotype and C allele of *IL23R* might increase GC risk. Moreover, gene-environment interactions among smoking status, family history of GC, and rs3748067 also contributed to GC risk. The expression of miR-10a-3p was upregulated in GC cells, and GenePharma-miR-10a-3p downregulated the expression of *IL17A* mRNA and protein in SGC-7901 cells. In addition, luciferase report gene assay proved miR-10a-3p target at *IL17A*-UTR. All the results illustrated that miRNA binding site SNPs within IL23/Th17 inflammatory pathway genes might influence gastric cancer risk.

## Supplementary Material

Supplemental Figure 1. The flow chart of the study experiments. The experiments mainly consist of two parts: association analysis for studying the associations between functional miRNA-binding sites single nucleotide polymorphisms (SNPs) with gastric cancer risk; functional study for preliminary verifying the regulatory role of miRNA on its corresponding positive SNPs.

## Figures and Tables

**Table 1:** SNPs of *IL17A*, *IL23R*, and *IL17RA* 3′-UTRs within miRNA binding sites.

Gene	SNP	MAF	Predicted miRNA binding	ΔΔ*G* (kJ/mol)
*IL17A*	rs3748067	0.13	hsa-miR-10a-3p	−9.59
*IL23R*	rs10889677	0.29	hsa-miR-1827	−19.12
			hsa-miR-5680	−10.37
*IL17RA*	rs1468488	0.08	hsa-miR-320a	−25.44
			hsa-miR-320b	−21.68
			hsa-miR-320c	−19.96
			hsa-miR-320d	−17.64
			hsa-miR-320e	−16.75
			hsa-miR-513c-5p	−19.47
			hsa-miR-514b-5p	−19.52
	rs887796	0.18	hsa-miR-4761-3p	−16.16

SNP: single-nucleotide polymorphism; MAF: minor allele frequency.

**Table 2:** PCR information and restriction enzymes used for genotyping of the four miRNA binding site SNPs.

Gene	SNP ID	Alleles	Annealing Tm (°C)	Restriction enzyme	Genotype (bp)	Primers
*IL17A*	rs3748067	C/T	58.2	ApoI	C: 317	Sense AGGATGGAGTGAAGAGGAA
					T: 202,115	Antisense AGAGATCAACAGACCAACAT
*IL23R*	rs10889677	A/C	56.6	MnlI	A: 165	Sense TGCTCCTACCATCACCAT
					C: 86,79	Antisense TGAGGCGTCCACATAATG
*IL17RA*	rs1468488	T/C	59.6	AluI	T: 164,94,92	Sense GGAGGAAGAGGAGGAAGAG
					C: 256,94	Antisense GGATAGACGATAACCAGACC
*IL17RA*	rs887796	A/G	56.6	BanI	A: 454	Sense AATTCTCCAAGGTGTCTGTT
					G: 263,191	Antisense GATCAAGATAGTAGGCAGGAA

SNP: single-nucleotide polymorphism.

**Table 3:** Demographic characteristics in GC patients and controls.

Variables	Cases (%)	Controls (%)	Test statistics	*P* ^b^	OR (95% CI)
	(*n* = 500)	(*n* = 500)			
Age (mean ± SD), year	57.93 ± 11.88	57.27 ± 12.15	*t* = 0.92	0.36^a^	
Gender					
Female	124 (24.80)	124 (24.80)			1
Male	376 (75.20)	376 (75.20)	*χ* ^2^ = 0.00	1.00	1.00 (0.75, 1.33)
Smoking status					
Never	202 (40.40)	255 (51.00)			1
Ever	298 (59.60)	245 (49.00)	*χ* ^2^ = 11.32	<0.01	1.53 (1.20, 1.97)
Drinking status					
Never	310 (62.00)	325 (65.00)			1
Ever	190 (38.00)	175 (35.00)	*χ* ^2^ = 0.97	0.32	1.14 (0.88, 1.47)
Family history of GC					
No	381 (76.20)	444 (88.80)			1
Yes	119 (23.80)	56 (11.20)	*χ* ^2^ = 27.49	<0.01	2.48 (1.75, 3.50)

GC: gastric cancer; OR: odds ratio; CI: confidence interval; SD: standard deviation. ^a^*P* value for Student's t-test. ^b^*P* value for two-sided *χ*^2^ test.

**Table 4:** Logistic regression analysis of associations between four miRNA binding site SNPs and GC risk.

	Cases (%)	Controls (%)	*P* ^a^	OR (95% CI)^b^	*P* ^b^	OR (95% CI)^c^	*P* ^c^
	(*n* = 500)	(*n* = 500)					
rs3748067							
CC	393 (78.60)	339 (67.80)		1.00		1.00	
CT	104 (20.80)	154 (30.80)		0.58 (0.44, 0.78)	<0.01	0.59 (0.44, 0.79)	<0.01
TT	3 (0.60)	7 (1.40)	0.07	0.37 (0.10. 1.44)	0.76	0.39 (0.10, 1.54)	0.18
TT + CT	107 (21.40)	161 (32.20)		0.57 (0.43, 0.76)	0.01	0.58 (0.43, 0.77)	<0.01
C	890 (89.00)	832 (83.20)		1.00		1.00	
T	110 (11.00)	168 (16.8)		0.61 (0.47, 0.79)	0.01	0.77 (0.47, 0.80)	<0.01
rs10889677							
AA	252 (50.40)	274 (54.80)		1.00		1.00	
AC	204 (40.80)	205 (41.00)		1.08 (0.84, 1.40)	0.55	1.07 (0.82, 1.39)	0.61
CC	44 (8.80)	21 (4.20)	0.10	2.28 (1.32, 3.94)	<0.01	2.22 (1.27, 3.87)	<0.01
CC + AC	248 (49.60)	226 (45.20)		1.19 (0.93, 1.53)	0.16	1.18 (0.92, 1.52)	0.20
A	708 (70.80)	753 (75.30)		1.00		1.00	
C	292 (29.20)	247 (24.70)		1.26 (1.03, 1.53)	0.02	1.24 (1.02, 1.52)	0.03
rs1468488							
TT	431 (86.20)	424 (84.80)		1.00		1.00	
CT	67 (13.40)	75 (15.00)		0.88 (0.62, 1.25)	0.48	0.82 (0.57, 1.18)	0.28
CC	2 (0.40)	1 (0.20)	0.47	1.97 (0.18, 21.78)	0.58	1.84 (0.17, 20.49)	0.62
CC + CT	69 (13.80)	76 (15.20)		0.91 (0.64, 1.29)	0.58	0.83 (0.58, 1.13)	0.32
T	929 (92.90)	923 (92.30)		1.00			
C	71 (7.10)	77 (7.70)		0.92 (0.66, 1.28)	0.61	0.86 (0.61, 1.21)	0.38
rs887796							
AA	385 (77.00)	387 (77.40)		1.00		1.00	
AG	104 (20.80)	101 (20.20)		1.04 (0.76, 1.41)	0.83	1.09 (0.80, 1.49)	0.59
GG	11 (2.20)	12 (2.40)	0.23	0.92 (0.40, 2.11)	0.85	0.94 (0.41, 2.18)	0.89
GG + AG	115 (23.00)	113 (22.60)		1.02 (0.76, 1.38)	0.88	1.08 (0.80, 1.45)	0.64
A	874 (87.40)	875 (87.50)		1.00		1.00	
G	126 (12.60)	125 (12.50)		1.01 (0.78, 1.32)	0.95	1.05 (0.80, 1.37)	0.72

GC: gastric cancer; SNP: single-nucleotide polymorphism; OR: odds ratio; CI: confidence interval. ^a^*P* value for Hardy-Weinberg equilibrium in controls. ^b^OR with 95% CI and *P* value for unadjusted logistic regression analysis. ^c^OR with 95% CI and *P* value for logistic regression analysis adjusted for smoking, drinking, and family history of GC.

**Table 5:** Stratification analysis of the four miRNA binding site SNPs and GC susceptibility.

Variables	rs3748067		rs10889677		rs1468488		rs887796	
	OR (95% CI)^a^	*P* ^a^	OR (95% CI)^a^	*P* ^a^	OR (95% CI)^a^	*P* ^a^	OR (95% CI)^a^	*P* ^a^
Age								
≤50	0.66 (0.39, 1.14)	0.14	1.29 (0.87, 1.92)	0.20	0.63 (0.30, 1.34)	0.23	1.25 (0.74, 2.12)	0.41
>50	0.57 (0.41, 0.78)	<0.01	1.24 (0.97, 1.58)	0.09	0.90 (0.60, 1.34)	0.60	0.97 (0.72, 1.32)	0.86
Gender								
Female	1.15 (0.46, 1.76)	0.77	0.94 (0.56, 1.56)	0.80	1.12 (0.43, 2.97)	0.82	1.08 (0.59, 1.98)	0.81
Male	0.55 (0.40, 0.75)	<0.01	1.29 (1.02, 1.65)	0.04	0.87 (0.58, 1.31)	0.51	1.08 (0.80, 1.47)	0.61
Smoking status								
Never	0.83 (0.55, 1.24)	0.35	1.16 (0.84, 1.60)	0.36	0.80 (0.46, 1.39)	0.42	1.07 (0.72, 1.58)	0.75
Ever	0.43 (0.29, 0.63)	<0.01	1.40 (1.05, 1.85)	0.02	0.94 (0.59, 1.49)	0.79	1.14 (0.79, 1.65)	0.47
Drinking status								
Never	0.60 (0.42, 0.86)	<0.01	1.29 (0.99, 1.68)	0.06	0.71 (0.46, 1.08)	0.11	0.85 (0.61, 1.18)	0.32
Ever	0.50 (0.31, 0.79)	<0.01	1.31 (0.92, 1.87)	0.13	1.52 (0.77, 2.99)	0.23	1.78 (1.12, 2.85)	0.02
Family history of GC								
No	0.58 (0.43, 0.79)	<0.01	1.33 (1.06, 1.66)	0.02	0.85 (0.58, 1.26)	0.42	0.99 (0.75, 1.32)	0.96
Yes	0.53 (0.26, 1.09)	0.08	1.06 (0.61, 1.84)	0.84	1.01 (0.42, 2.45)	0.98	2.01 (0.86, 4.67)	0.11

GC: gastric cancer; SNP: single-nucleotide polymorphism; OR: odds ratio; CI: confidence interval. ^a^OR with 95% CI and *P* value for logistic regression analysis adjusted for smoking, drinking, and family history of GC.

**Table 6:** Haplotype analysis of rs1468488 and rs887796 polymorphism sites in *IL17RA.*

Haplotype	Cases (%)	Controls (%)	*χ* ^2^	OR_adj_ (95% CI)^a^	*P* ^a^
Hap1 (*C*_rs1468488_*A*_rs887796_)	69 (6.90)	67 (6.70)	0.04	1.04 (0.73, 1.47)	0.84
Hap2 (*T*_rs1468488_*A*_rs887796_)	806 (80.60)	807 (80.70)	0.02	1.02 (0.81, 1.27)	0.90
Hap3 (*T*_rs1468488_*G*_rs887796_)	117 (11.70)	122 (12.20)	0.10	0.96 (0.73, 1.26)	0.76

OR: odds ratio; CI: confidence interval. ^a^OR with 95% CI and *P* value for logistic regression analysis adjusted for smoking, drinking, and family history of GC.

**Table 7:** Combined effect of the four miRNA binding site SNPs on GC risk.

Combined effect of risk alleles^a^	Cases (%)	Controls (%)	*χ* ^2^	*P*_trend_	OR (95% CI)
0-1	328 (65.60)	320 (64.00)			1.00
2-3	161 (32.20)	170 (34.00)	0.34	0.56	0.92 (0.71, 1.20)
4-5	11 (2.20)	10 (2.00)	0.03	0.87	1.07 (0.45, 2.56)
			0.18	0.67	
Total	500 (100)	500 (100)			

GC: gastric cancer; SNP: single-nucleotide polymorphism; OR: odds ratio; CI: confidence interval. ^a^The risk alleles: rs3748067T, rs10889677C, rs1468488C, and rs887796G.

**Table 8:** MDR models of selected polymorphisms and environmental risk factors.

Model	TBA^b^	CVC^c^	*χ* ^2^	OR (95% CI)	*P*
FH^a^	0.56	10/10	27.49	2.48 (1.75, 3.50)	<0.01
Smoking, rs3748067	0.57	9/10	30.53	2.05 (1.59, 2.65)	<0.01
Smoking, FH^a^, and rs3748067	0.59	9/10	40.00	2.25 (1.75, 2.90)	<0.01

MDR: multifactor dimensionality reduction; OR: odds ratio; CI: confidence interval. ^a^Family history of GC. ^b^Testing balance accuracy. ^c^Cross-validation consistency.
