# Supplementary material for: Associations of Functional MicroRNA Binding Site Polymorphisms in IL23/Th17 Inflammatory Pathway Genes with Gastric Cancer Risk
Source: Mediators Inflamm. 2017 Oct 8;2017:6974696. doi: 10.1155/2017/6974696 (PMC5651119; doi:10.1155/2017/6974696)
Supplement: Supplementary file 1 — Supplemental Figure 1. The flow chart of the study experiments. The experiments mainly consist of two parts: association analysis for studying the associations between functional miRNA-binding sites single nucleotide polymorphisms (SNPs) with gastric cancer risk; functional study for preliminary verifying the regulatory role of miRNA on its corresponding positive SNPs. [file 6974696.f1.ppt]

## Slide 1
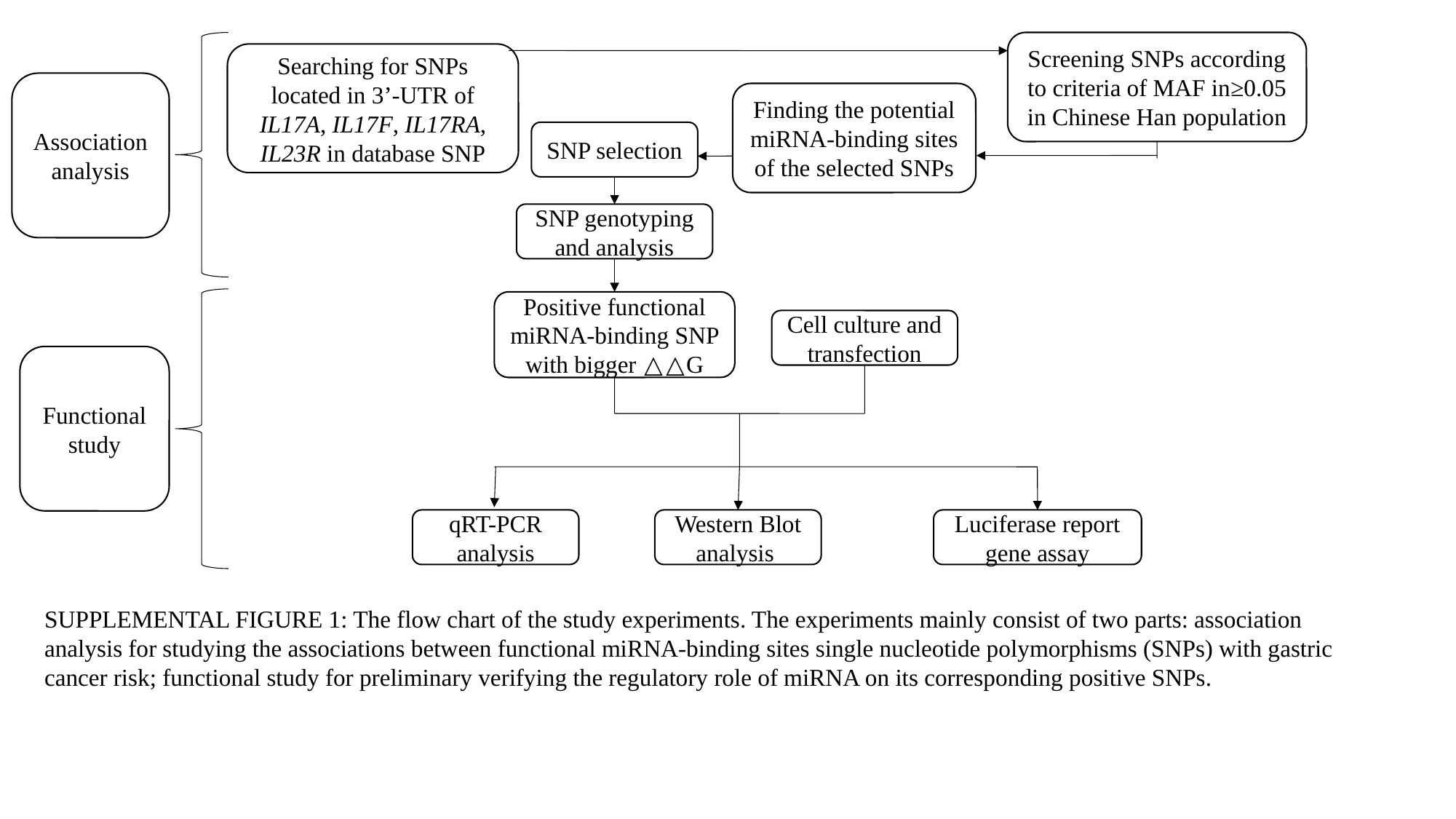

Screening SNPs according to criteria of MAF in≥0.05 in Chinese Han population
Searching for SNPs located in 3’-UTR of IL17A, IL17F, IL17RA, IL23R in database SNP
Association analysis
Finding the potential miRNA-binding sites of the selected SNPs
SNP selection
SNP genotyping and analysis
Positive functional miRNA-binding SNP with bigger △△G
Cell culture and transfection
Functional study
qRT-PCR analysis
Western Blot analysis
Luciferase report gene assay
SUPPLEMENTAL FIGURE 1: The flow chart of the study experiments. The experiments mainly consist of two parts: association analysis for studying the associations between functional miRNA-binding sites single nucleotide polymorphisms (SNPs) with gastric cancer risk; functional study for preliminary verifying the regulatory role of miRNA on its corresponding positive SNPs.
